# Toe Nails Inverted

**Published:** 1857-04

**Authors:** 


### TOE NAILS INVERTED.

SOME years ago we had a lady under our care. Having been raised in luxury and wealth, we found it impracticable to secure the amount of daily out-door exercise which the symptoms of her case seemed to require. By dint of cross questions, however, we ascertained that she was unable to walk much, in consequence of an inverted toe nail.

"But why did you not let me know that before?"

"I visited New York a year ago, to consult one of your celebrated men. He split the nail, and dragged a part of it out by main force. The pain was terrible, and I determined I would rather die than submit to a second operation, especially as it grew out again, and is now so painful that a large portion of my time I can scarcely walk."

We assured her that it could most probably be cured without the slightest pain whatever. We advised her to take a piece of broken window-glass and a small bit of raw cotton, and employ them according to the usage of experienced physicians. She was utterly incredulous, but followed our directions, and in a very short time could walk well without discomfort; and, at the end of five years, her husband writes, under date of February 17th, 1857, "Her toe is perfectly well, cured entirely by your simple prescription." Moral: *Keep nothing from your physician.*

